# Primary Rhabdomyosarcoma of the Pleura: A Case Report and Review of the Literature

**DOI:** 10.7759/cureus.12491

**Published:** 2021-01-05

**Authors:** Nabila Chekhlabi, Jihane Toughza, Nezha Dini

**Affiliations:** 1 Pediatric Department, International University Hospital Cheikh Khalifa, Mohammed VI University of Health Sciences, Casablanca, MAR; 2 Pediatric Oncology Department, International University Hospital Cheikh Khalifa, Mohammed VI University of Health Sciences, Casablanca, MAR; 3 Pediatric Department, Faculty of Medicine and Pharmacy, Mohammed V University, Rabat, MAR

**Keywords:** rhabdomyosarcoma, pleural effusion, child

## Abstract

Rhabdomyosarcoma is a malignant tumor of striated muscle tissue that can exceptionally present in the pleura. Its prognosis is generally poor. We report a case of an eight-year-old child admitted for a persistent dry cough which had progressed for three weeks, complicated by breathing difficulties and a deterioration in general condition. He had a history of recent contact with an uncle with pulmonary tuberculosis. Clinical examination on admission revealed right pleural effusion syndrome with moderate respiratory repercussion. The biological assessment shows moderate inflammation and a very high level of lactate dehydrogenase (LDH). Radiologically, there was abundant right pleurisy with intra-pleural nodular masses without ipsilateral pulmonary invasion or other distant localization. Pleural puncture reveals exudative lymphocytic fluid with negative tuberculous polymerase chain reaction (PCR) and atypical cells in cytology. Biopsy of the pleural mass showed pleural rhabdomyosarcoma of the alveolar type. The pet scan found bone metastases at two costal arches. After conditioning, the child received several courses of chemotherapy. The clinical and radiological outcome was favorable. This case is reported in view of its rarity and originality. We conclude that early diagnosis and treatment greatly improves the prognosis of this aggressive tumor.

## Introduction

Rhabdomyosarcoma (RMS) is a malignant tumor of striated muscle tissue. It is mainly located in the head, the extremities of the limbs and the urogenital sinus, while it rarely affects the thorax and exceptionally the pleura. Embryonic RMS, the most common in children, has a better prognosis compared to the alveolar type. Only a few cases of primary pleural rhabdomyosarcoma have been reported in the literature. We report a case, while discussing the clinical aspects, treatment and prognosis of this localization.

## Case presentation

This is an eight-year-old child with a history of recent tuberculosis contagion, admitted with a dry and painful cough progressing over a three-week period before admission, complicated by breathing difficulties, fever, and worsening of the condition in general. The clinical examination, on admission, found an asthenic child, febrile at 39°, in respiratory distress with a respiratory rate of 52 breaths per minute and saturation at 92%. Pleuropulmonary examination revealed a massive right-sided pleural effusion. The remainder of the examination did not find locoregional lymphadenopathy or hepatosplenomegaly. The biological workup revealed a normal blood count and a moderate increase in inflammatory markers with an erythrocyte sedimentation rate (ESR) of 42 mm and a C reactive protein (CRP) of 34 mg/l. The level of lactate dehydrogenase (LDH) was elevated to 1345 IU/L. The quantiferon test for tuberculosis was negative.

**Figure 1:**
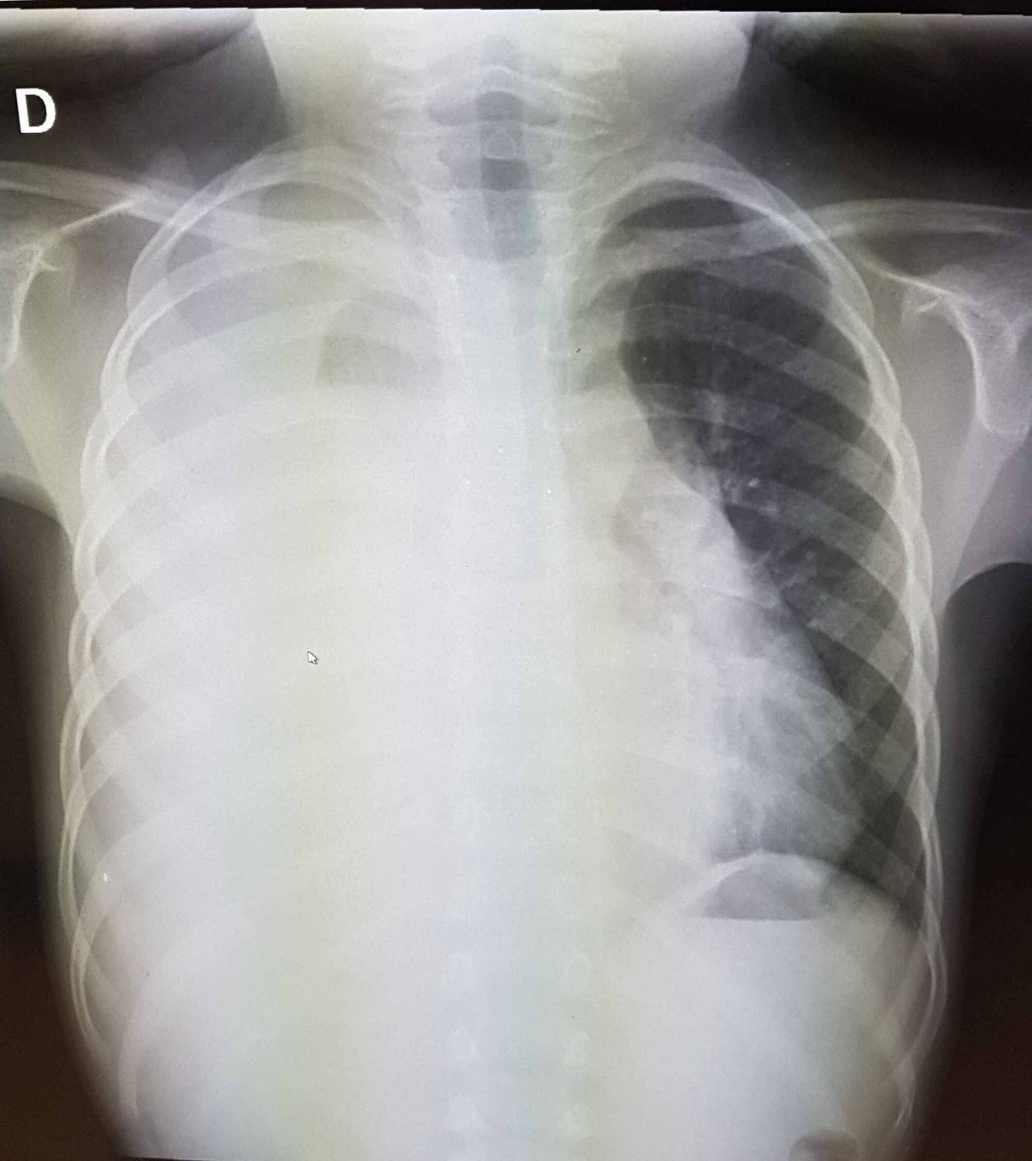
Standard chest x-ray showing massive right pleurisy

**Figure 2:**
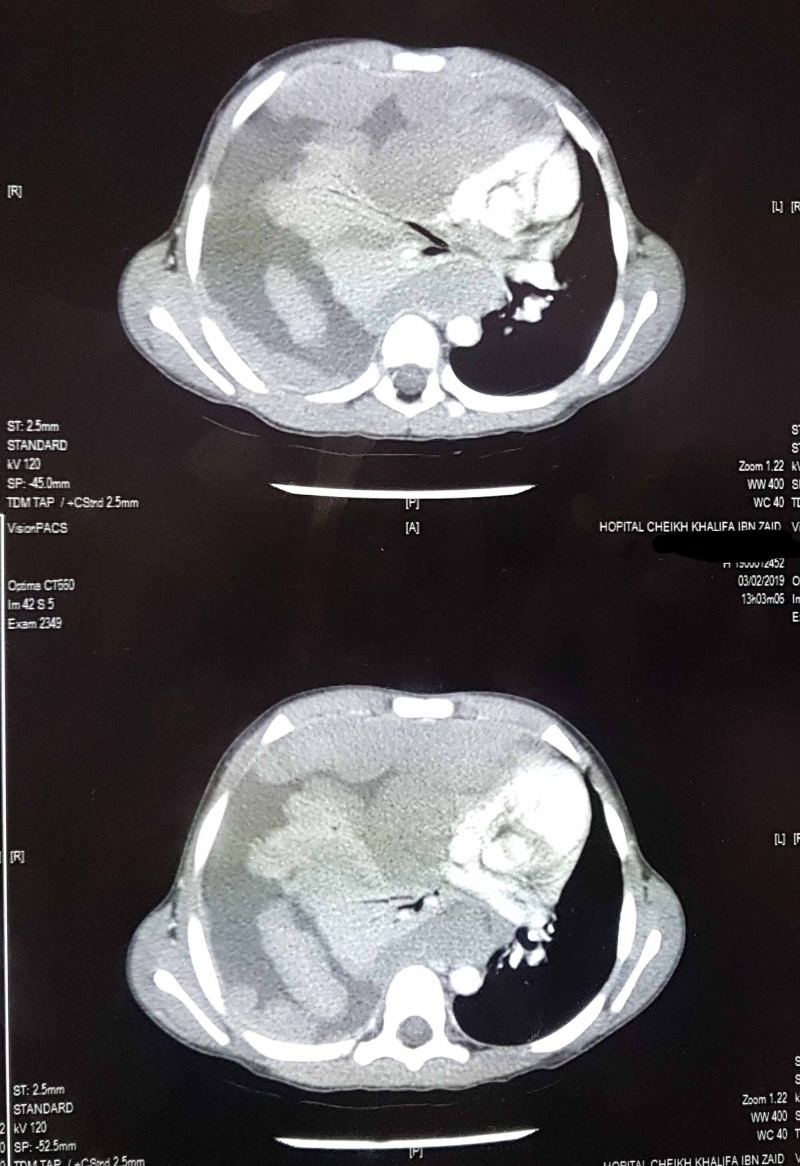
Axial CT sections of the thorax: Right pleurisy with polylobed intra-pleural masses

Radiologically, a standard chest X-ray followed by a chest CT scan showed massive right pleurisy with intra-pleural nodular masses without ipsilateral pulmonary invasion or other distant localization (Figure 1, 2).

**Figure 3:**
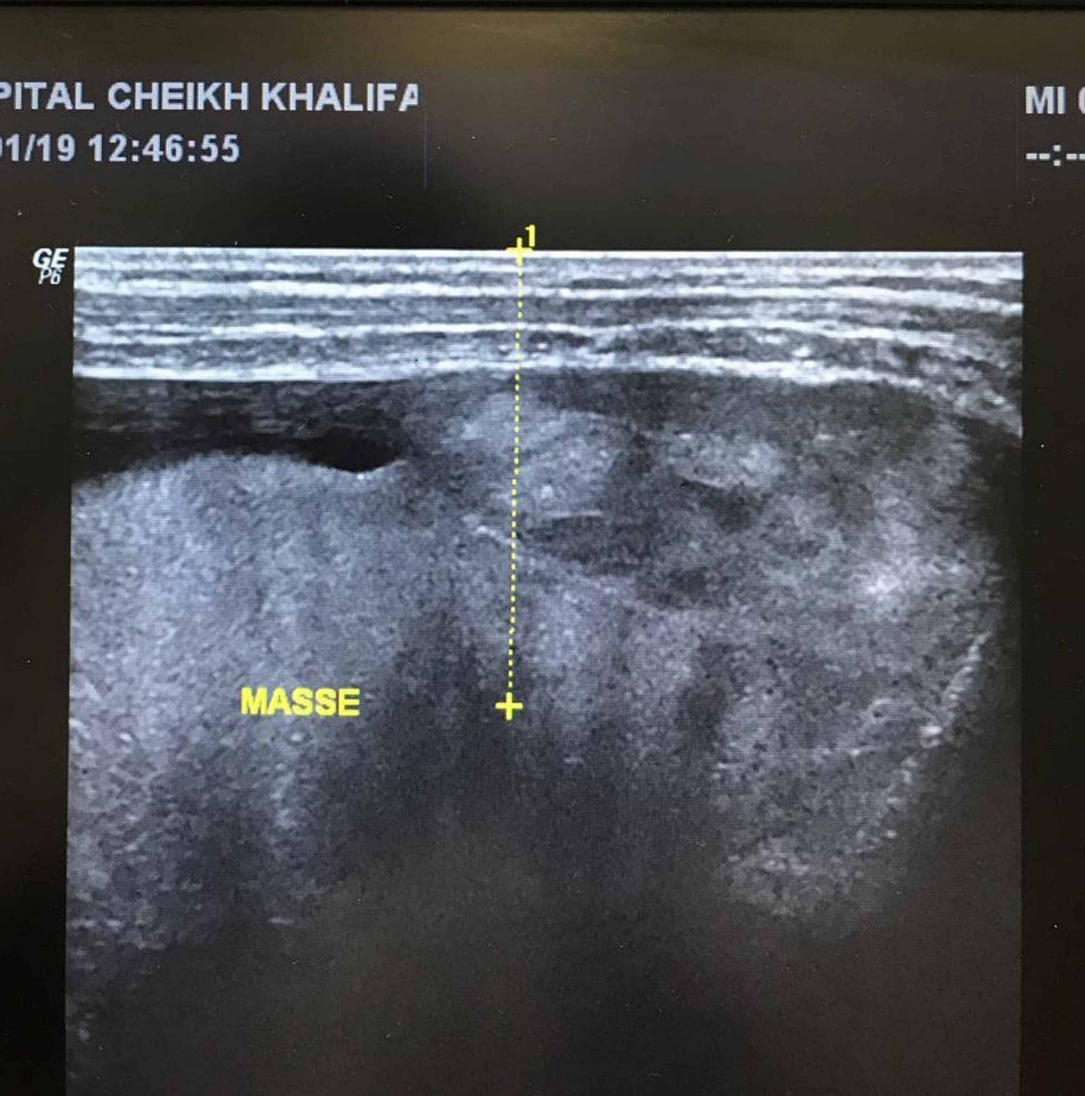
Chest ultrasound showing a multinodular mass and guiding the puncture-biopsy.

Pleural puncture revealed exudating serohematic fluid with lymphocyte cellularity, a negative polymerase chain reaction (PCR) test for tuberculosis, and atypical cells in the cytological examination. An ultrasound-guided puncture-biopsy of the pleural mass was performed under local anesthesia (Figure 3).

**Figure 4:**
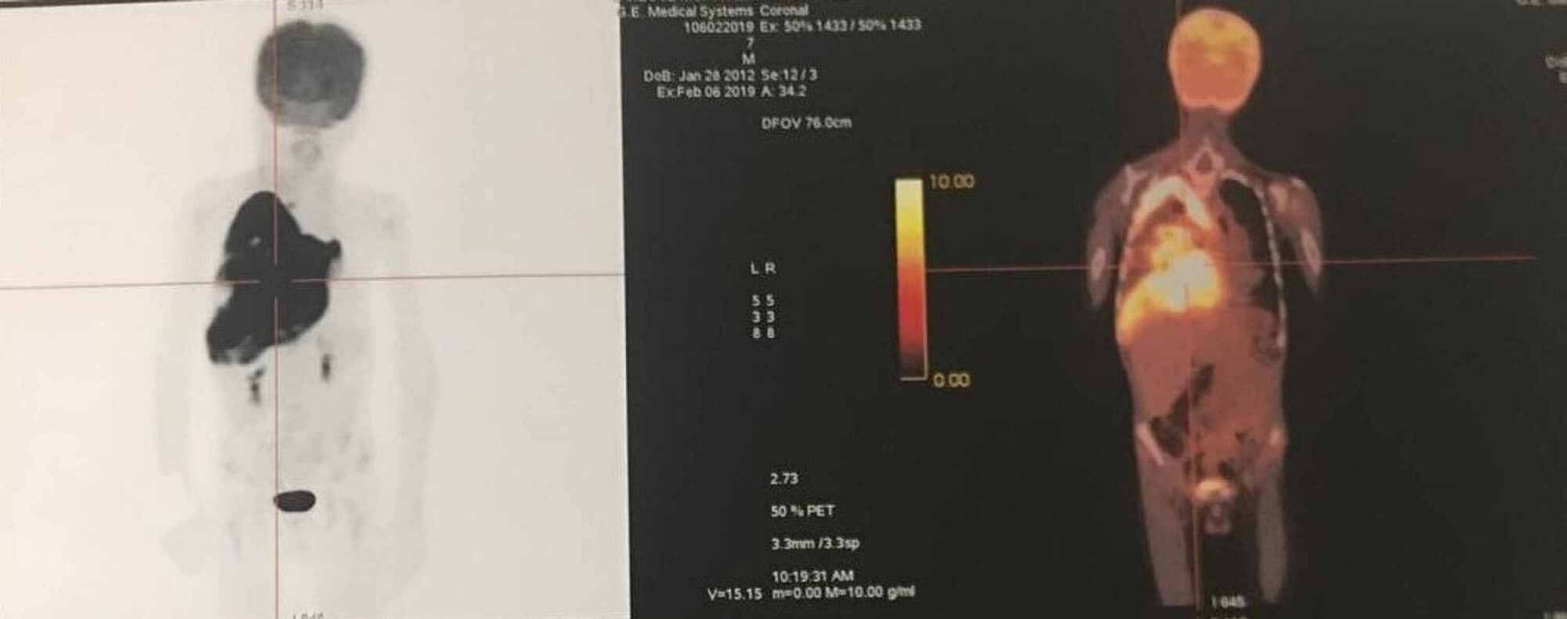
The positron emission tomography/computed tomography (PET/CT) detects a hyper-metabolic zone of the entire right hemitorax.

The histopathological examination of the biopsy found a tumor process composed of cells of medium to large size, with hyperchromatic anisokaryotic nuclei, located in an eosinophilic cytoplasm of rhabdoid appearance. Immunohistochemistry shows strong and diffuse positivity for anti-desmin and anti-myogenin muscle antibodies. This aspect was in favor of a pleural rhabdomyosarcoma of the alveolar type. The positron emission tomography/computed tomography (PET/CT) detected a hyper-metabolic zone of the entire right hemitorax pushing the mediastinal structures to the left with the invasion of the right posterior arch (Figure 4).

A bone marrow biopsy and bone scintigraphy showed no abnormalities. The patient was started on a conditioning regimen followed by several courses on chemotherapy. Clinical and radiological evaluation at three months showed complete resolution of the pleural masses and pleural effusion. During a 12-month follow-up, the patient's condition was stable with complete clinical and radiological remission.

## Discussion

Among the malignant mesenchymal tumors in children, rhabdomyosarcoma (RMS) is the most common. It is considered a childhood disease because the vast majority of cases occur in people under the age of 18 [1]. RMS is slightly more common in boys with a sex ratio of 1.4. It mainly affects the head and neck (35%), more rarely the urogenital system (21%), the extremities (19%), and the chest (7%) [1]. Primary involvement of the pleura is exceptional. To our knowledge, only a few cases of pleural rhabdomyosarcoma have been reported in the literature [1]. Indeed, the first case was documented by Hamada et al. in 1989 [2].

Thoracic RMS remains asymptomatic for a long time; it becomes symptomatic when the tumor reaches a considerable size. In case of isolated pleural location, pleurisy gradually increasing in size is the most reported revealing sign in the literature. The radiological appearance of thoracic RMS is not specific in standard chest x-ray; it often shows pleurisy of variable abundance. The chest CT scan shows a generally multiple masses, large in size and varying in density depending on possible cystic and/or necrotic changes in the tumor [1].

In our case, the diagnosis was initially unclear due to a history of close contact to an uncle with infectious tuberculosis. Pleuropulmonary tuberculosis was considered in our patient, given the deterioration of the general condition, the fever and the radioclinical pleural effusion syndrome. However, the laboratory workup proved otherwise, with an elevated lactate dehydrogenase (LDH) level, negative tests for tuberculosis (quantiferon and PCR tests). Atypical cells were also seen in the pleural fluid which made a neoplastic cause more probable. A definitive diagnosis of pleural RMS was reached by chest computed tomography and histopathological examination of pleural biopsy.

RMS are very aggressive malignant mesenchymal tumors that arise from immature cells intended to form skeletal striated muscle [3]. The characteristic cells of this tumor are the rhabdomyoblasts. They are slightly elongated with intracellular cross streaks and eosinophilic cytoplasm. These very specific streaks and the elongated or spindle-shaped appearance of the cell with several nuclei are signs of myoblastic maturity found in 50 to 60% of embryonic subtypes and 30% of alveolar subtypes [4]. There are four histological subtypes: embryonic, botryoid, alveolar and pleomorphic. The embryonic type, most common in children, has a better prognosis compared to alveolar RMS, while the latter is known for its great aggressiveness. An immunohistochemical study is necessary to confirm the diagnosis. Nuclear labeling with MyoD1 and myogenin, indicating striated muscle differentiation, is specific for RMS. In addition, tumor cells show frequent positivity for desmin [5]. The expression of myogenin differs between the subtypes and constitutes a good element of histological classification: the labeling is diffuse in alveolar RMS (up to 100% of tumor cells) and is much more heterogeneous in embryonic RMS, affecting usually less than two-thirds of the cells [6].

Thoracic and abdominal imaging, bone scintigraphy and bone marrow biopsy are an integral part of the clinical staging workup [7]. Positron emission tomography/computed tomography (PET/CT) provides very accurate data in assessing the extent of RMS [8].

Effective treatment of RMS requires a combination of surgery, chemotherapy, and radiation therapy. Chemotherapy aims to reduce the tumor size as much as possible to make it accessible for surgery. In cases diagnosed early, chemotherapy alone may be enough to make the tumor completely disappear. A residual tumor mass after surgery negatively influences overall survival. Surgical resection with healthy margins can avoid additional radiation therapy and therefore prevent the long-term side effects of radiation therapy [9]. Chest radiotherapy has numerous side effects including pulmonary fibrosis, scoliosis, cardiomyopathy and a significant risk of secondary malignancies. Therefore, this therapeutic approach should be chosen after careful patient selection [10]. Moreover, *Berberis orthobotrys* is a Pakistani medicinal plant that has long been used in the traditional treatment of cancers. A large study from Pakistan provides the first evidence that treatment with this herb is effective against RMS cancer cells with few side effects on healthy cells [11].

Most rhabdomyosarcomas do not have inherited family factors. There is therefore no reason to test the parents, brothers, or sisters of a child with rhabdomyosarcoma. Our patient had no family history of malignancy, including rhabdomyosarcoma. In addition, favorable prognostic factors are age at diagnosis less than 10 years, maximum tumor extent less than 5 cm, embryonic type on histology and location of the tumor [12]. The pleural site is generally considered a poor prognostic factor when compared with other locations, because of its higher metastatic potential, the difficulty of local treatment, and the higher risk of local recurrence [13].

## Conclusions

Rhabdomyosarcoma is a very aggressive malignant tumor originating in the striated muscle, usually located in the head and neck, genitourinary tract, and extremities. Although primary pleural rhabdomyosarcoma is extremely rare, it should be considered in cases of non-infectious pleurisy for early detection and treatment. Prompt diagnosis can improve the overall prognosis of this condition and its responsiveness to chemotherapy.
